# IL-27 inhibits epithelial-mesenchymal transition and angiogenic factor production in a STAT1-dominant pathway in human non-small cell lung cancer

**DOI:** 10.1186/1756-9966-32-97

**Published:** 2013-11-25

**Authors:** Puja Kachroo, Mi-Heon Lee, Ling Zhang, Felicita Baratelli, Gina Lee, Minu K Srivastava, Gerald Wang, Tonya C Walser, Kostyantyn Krysan, Sherven Sharma, Steven M Dubinett, Jay M Lee

**Affiliations:** 1 Lung Cancer Research Program, Jonsson Comprehensive Cancer Center, Los Angeles, CA, USA; 2 Division of Pulmonary and Critical Care Medicine, Los Angeles, CA, USA; 3 Division of Thoracic Surgery at the David Geffen School of Medicine, University of California, Los Angeles, CA, USA; 4 Molecular Gene Medicine Laboratory, Veterans Affair Greater Los Angeles Healthcare System, Los Angeles, CA, USA; 5 Division of Thoracic Surgery, Ronald Reagan UCLA Medical Center, Room 64-128 CHS, 10833 Le Conte Ave, Box 957313, Los Angeles, CA 90095-7313, USA

**Keywords:** IL-27, STAT1, STAT3, Epithelial-mesenchymal transition, Cytokine, Angiogenesis

## Abstract

**Background:**

Interleukin-27 signaling is mediated by the JAK-STAT pathway via activation of STAT1 and STAT3, which have tumor suppressive and oncogenic activities, respectively. Epithelial–mesenchymal transition (EMT) and angiogenesis are key processes in carcinogenesis. Although IL-27 has been shown to have potent anti-tumor activity in various cancer models, the role of IL-27 in EMT and angiogenesis is poorly understood. In this study, we investigated the role of IL-27 in regulating EMT and angiogenesis through modulation of the STAT pathways in human non-small cell lung carcinoma (NSCLC) cells.

**Methods:**

STAT activation following IL-27 exposure was measured in human NSCLC cell lines. Expression of epithelial (E-cadherin, γ-catenin) and mesenchymal (N-cadherin, vimentin) markers were assessed by Western blot analysis. Production of pro-angiogenic factors (VEGF, IL-8/CXCL8, CXCL5) were examined by ELISA. Cell motility was examined by an *in vitro* scratch and transwell migration assays. Selective inhibitors of STAT1 (STAT1 siRNAs) and STAT3 (Stattic) were used to determine whether both STAT1 and STAT3 are required for IL-27 mediated inhibition of EMT and secretion of angiogenic factors.

**Results:**

Our results demonstrate that IL-27 stimulation in NSCLC resulted in 1) STAT1 and STAT3 activation in a JAK-dependent manner, 2) development of epithelial phenotypes, including a decrease in the expression of a transcriptional repressor for E-cadherin (SNAIL), and mesenchymal marker (vimentin) with a reciprocal increase in the expression of epithelial markers, 3) inhibition of cell migration, and 4) reduced production of pro-angiogenic factors. STAT1 inhibition in IL-27–treated cells reversed the IL-27 effect with resultant increased expression of Snail, vimentin and the pro-angiogenic factors. The inhibition of STAT3 activation had no effect on the development of the epithelial phenotype.

**Conclusion:**

IL-27 induces mesenchymal to epithelial transition and inhibits the production of pro-angiogenic factors in a STAT1–dominant pathway. These findings highlight the importance of STAT1 in repressing lung carcinogenesis and describe a new anti-tumor mechanism of IL-27.

## Background

Interleukin-27 (IL-27) is a member of the IL-12 cytokine family known to have both pro-inflammatory and anti-inflammatory functions [1]. In preclinical models, IL-27 has been shown to have anti-tumor properties in a variety of malignancies through several mechanisms, including inhibition of tumor proliferation and angiogenesis [2-8]. IL-27 has attracted interest as an anti-tumor agent because of its similarities to IL-12, which also demonstrated ability to suppress tumor growth and elicit tumor specific immune responses [9]. However, the use of IL-12 as a single agent has been limited by its toxicity and poor response in clinical trials for advanced renal or ovarian cancers necessitating studies in other promising agents [9,10].

IL-27 elicits its effects through activation of both STAT1 and STAT3, which have opposing roles in carcinogenesis [1,2,8,11-15]. Activated STAT1 signaling has tumor suppressive roles by inhibiting angiogenesis, tumor growth and metastasis as well as promoting apoptosis [12,16]. Alternatively, the STAT3 pathway has been shown to be constitutively activated in many human cancers and has been implicated in oncogenic transformation and progression [17-21]. IL-27 is a heterodimeric molecule, composed of Epstein-Barr virus-induced gene 3 (EBI3) and p28 subunits, that is expressed by activated antigen presenting cells [22]. The intracellular component of its receptor, comprised of glycoprotein 130 (gp130) and WSX-1 (also known as IL-27Rα or TCCR), associates with cytoplasmic protein kinases such as JAKs (Janus Activated Kinases) that mediate cytokine signaling [1]. The JAK-Signal Transducer and Activator of Transcription (STAT) signaling pathway, which was initially identified as a critical process in normal cellular processes, has also been implicated in tumor initiation and malignant progression. The STAT transcriptional factors, which are phosphorylated by the JAKs, dissociate from the receptor and dimerize followed by nuclear translocation [23].

Epithelial-mesenchymal transition (EMT) is an evolutionarily conserved process in which cells undergo conversion from an epithelial to mesenchymal phenotype whereby cells develop loose cell-cell interactions and become motile [24]. The importance of EMT in driving carcinogenesis has been shown in lung, breast, prostate, pancreatic, and liver cancers [25,26].

IL-27 mediated inhibition of angiogenesis is a known anti-tumor mechanism in various malignancies [3,5]. Although a study showed that either over-expression or treatment with recombinant IL-27 led to anti-tumor activity on murine and human lung cancer cells, there is limited insight on the mechanism that modulates EMT and angiogenesis [27]. Furthermore, the mechanisms by which IL-27 plays a role in modulation of EMT and angiogenesis in NSCLC through the STAT pathways have not been studied. On this basis and given the fact that IL-27 regulates STAT transcriptional factors (STAT1 and STAT3) that possess opposing activities in cancer, the impact of this cytokine on lung carcinogenesis was investigated. Here, we report that IL-27 promotes the expression of epithelial markers, inhibits cell migration and the production of angiogenic factors in human NSCLC through a STAT1 dominant pathway. To our knowledge, the antitumor activity of IL-27 through a STAT1 dependent pathway has not been previously described.

## Materials and methods

### Cell lines and culture

Human NSCLC cell lines (A549, H2122, H1703, H292, H1437, H460, H1650, and H358) were obtained from the American Type Culture Collection (Rockville, MD). The H157 cell line was obtained from the National Cancer Institute (Bethesda, MD). Cells were verified by genotyping and tested for *Mycoplasma*. The cancer cells lines were maintained in RPMI-1640 with L-glutamine (Hyclone, Logan, UT) supplemented with 5% fetal bovine serum (FBS; Gemini Bio-products, West Sacramento, CA) in a humidified atmosphere of 5% CO_2_ at 37°C.

### Reagents

Recombinant human IL-27 (R&D Systems, Inc, Minneapolis, MN) was added at a concentration of 50 ng/mL in serum-free medium. JAK inhibitor I (Santa Cruz Biotechnology, Inc., Santa Cruz, CA) binds to the JAK2 kinase domain and inhibits JAK1, JAK2, and JAK3. It was reconstituted in DMSO and added at various concentrations from 1-100 nM in serum-free medium. STAT3 inhibitor V, Stattic (Santa Cruz Biotechnology, Inc, Santa Cruz, CA), is a nonpeptidic small molecule that selectively inhibits the SH2 domain of STAT3, thereby blocking its phosphorylation and dimerization. It was dissolved in DMSO and used at a concentration of 7.5 nM in serum-free medium. Opti-MEM I Reduced Serum-Medium and Lipofectamine 2000 reagents (Invitrogen, Carlsbad, CA) were utilized for transfection.

### Flow cytometry

A549 cells were stained with anti-human IL-27 Rα/WSX-1/TCCR-PE or isotype control (R&D systems, Minneapolis, MN) for 30 min at room temperature and analyzed by FACSCalibur (BD, San Jose, CA). FACS data were analyzed using Flowjo software (Treestar, Ashland, OR).

### Transfection of STAT1 small interfering RNA into A549 cells

Cells were seeded in 6-well plates and grown to 40-50% confluence at the time of transfection. For each sample, 2.5 μL of siRNA (10 μM) was diluted in 200 μL of Opti-MEM I. Two different constructs of STAT1 siRNA (Cell Signaling Technology, Danvers, MA) were used to inhibit STAT1 and a non-targeting siRNA (Ambion, Carlsbad, CA) was used as a control siRNA. The sequences for the STAT1 siRNAI and STAT1 siRNAII are 5’-CGAGAGCUGUCUAGGUUAAC-3' and 5'- GGGCAUCAUGCAUCUUACU-3', respectively. Similarly, 2.5 μL of Lipofectamine 2000 was diluted in 200 μL of Opti-MEM I. After 5 minutes of incubation at room temperature, the diluted oligomers were combined with the diluted Lipofectamine 2000 and incubated for 30 minutes at room temperature. The oligomer-Lipofectamine 2000 complexes were then added to each well containing the cells and medium and mixed gently. The cells were then incubated at 37°C in a CO_2_ incubator for 6 hours after which the wells were washed and further cultured for 18 hours after replaced with serum-free medium. The cells were then treated with IL-27 and/or Stattic per experimental design.

### Western blot

Cell lysates were prepared with RadioImmunoPrecipitation Assay (RIPA) buffer (PBS, 1% NP-40, 0.5% Na-deoxycholate, 0.1% SDS) containing protease inhibitors on ice after washing with PBS and were centrifuged at 13,000 rpm for 20 minutes at 4°C. Protein concentrations of cell lysates were measured by BCA assay and up to 20 μg of total protein were used for each SDS-PAGE. Western blot was performed after transferring SDS-PAGE gels to Amersham Hybond-ECL membranes (GE Healthcare, Piscataway, NJ). After incubation with 5% nonfat milk or BSA in TBST (10 mM Tris, pH 8.0, 150 mM NaCl, 0.5% Tween 20) for 1 hour at room temperature, the membrane was incubated with antibodies against phosphorylated-STAT1 (Tyr 701,1:1000), total-STAT1(1:1000), phosphorylated-STAT3 (Tyr 705, 1:1000 dilution), total-STAT3 (1:1000 dilution), Snail (1:1000) (Cell Signaling Technology, Danvers, MA), and Vimentin (1:2000) (BD Biosciences, San Jose, CA) at 4°C for overnight, and N-cadherin (1:5000), γ-catenin (1:7000), E-cadherin (1:6000), (BD Biosciences, San Jose, CA), and GAPDH (1:10,000) (Advanced ImmunoChemical, Long Beach, CA) at room temperature at 1 hour. Membranes were washed three times for 10 min and incubated with a 1:10,000 dilution of horseradish peroxidase-conjugated anti-mouse or anti-rabbit antibodies (Santa Cruz Biotechnology, Dallas, Texas). Blots were washed with TBST three times and developed with the ECL system (PerkinElmer, Waltham, MA) according to the manufacturer’s protocols.

### Enzyme-linked immunosorbent assay (ELISA)

ELISA kits for human vascular endothelial growth factor (VEGF), IL-8/CXLC8, and CXCL5 were used (R&D Systems, Minneapolis, MN). Concentrations of human VEGF, IL-8/CXCL8 and CXCL5 in culture supernatant were measured by ELISA following kit instructions. Briefly, 100 μL of the samples were loaded on the plates and incubated for 2 hours at room temperature. After the plates were washed with wash buffer (0.05% Tween20 in PBS), they were incubated with detection antibody for 2 hours at room temperature. Immunoreactivity was determined by adding substrate solution and absorbance (450 nm) was determined by Vmax Kinetic microplate reader (Molecular Devices, Sunnyvale, CA).

### In vitro cell motility assay

Cancer cells were plated in 6-well flat-bottom plates and allowed to adhere overnight. After serum starvation, cells were subject to different treatment conditions. Once the cells reached 90-95% confluence, a 200 μL pipette tip was used to make a scratch in the monolayer of cells in each well. The same fields were observed for cell migration using a phase-contrast microscope and photographed at various time points for up to 60 hours.

### Transwell cell migration assay

Cell migration assay was performed using a 96 well transwell chamber (Corning, Corning, NY). Cells were treated with STAT1 siRNAII (Cell Signaling Technology, Danvers, MA) for 24 hours and/or Stattic for 1 hour prior to adding IL-27. At 1 day of IL-27 treatment, 2 × 10^4^ cells in 75 ul were added to the bottom chamber of a 96-well plate with 8 μm pore size insert. Cells were allowed to transmigrate into the lower chamber containing 150 ul of RPMI/10% FBS. The non-migratory cells on the upper chamber surface were removed, and the upper and lower chambers were washed with PBS. After washing, 200 ul of Cell dissociation solution (Cultrex, Kampenhout, Belgium) containing Calcein AM (final 1.67 uM) (Molecular Probes, Eugene, OR) was added to the bottom chamber before reassembling the upper chamber. The plate was incubated at 37°C in CO_2_ incubator for 1 hour. At the end of incubation, the upper chamber was removed and the plate was read at 485 nm excitation for excitation and 520 nm for emission using the FLx800 fluorescence reader (BioTek, Winooski, Vermont). For maximum cell migration (100%) and background control, same amount of cells and medium, respectively, were directly added to the bottom chamber. Migration rate was calculated using the following formula:

$$\begin{array}{l} \mathrm{migration}\;\mathrm{rate}\;\left(\%\right)=\{\left(\mathrm{Mean}\;\mathrm{of}\;\mathrm{fluorescence}\;\mathrm{of}\;\mathrm{test}\;\mathrm{wells}‒\mathrm{Mean}\;\mathrm{of}\;\mathrm{fluorescence}\;\mathrm{of}\;\mathrm{background}\;\mathrm{control}\;\mathrm{wells}\right)\\ /\left(\mathrm{Mean}\;\mathrm{of}\;\mathrm{fluorescence}\;\mathrm{of}\;\mathrm{maximum}\;\mathrm{migration}\;\mathrm{wells}‒\mathrm{Mean}\;\mathrm{of}\;\mathrm{fluorescence}\;\mathrm{of}\;\mathrm{background}\;\mathrm{control}\;\mathrm{wells}\right)\}\\ \times 100. \end{array}$$

### Immunofluorescence

A549 cells were cultured to 40-60% confluence on glass coverslips (ThermoFisher Scientific, Waltham, MA), allowed to adhere overnight, and placed in serum free medium for four hours prior to IL-27 exposure for 15 minutes at 37°C. The cells were fixed with 4% paraformaldehyde (Electron Microscopy Sciences, Hatfield, PA) for 20 minutes at room temperature and then permeabilized with methanol for 15 minutes at -20°C. After blocking with 5% BSA in PBS solution for 1 hour at room temperature, the coverslips were incubated with primary antibody (1:100 dilution) overnight at 4°C. The following day, the coverslips were incubated with fluorescein-conjugated goat anti-rabbit IgG secondary antibody (1:50 dilution; Jackson ImmunoResearch Laboratories, Inc., West Grove, PA) for 30 minutes at room temperature followed by the addition of a DAPI (4'-6-Diamidino-2-phenylindole) nuclear stain (1:2000 dilution) for 2 minutes at room temperature. ProLong Gold antifade reagent (Invitrogen) was placed on the coverslip and the cells were then observed under the microscope. The ImageJ (Image Processing and Analysis in Java) program was used to create the merged immunofluorescence images.

### Statistics

Statistical significance was determined using the two-tailed Student’s *t* test and *p* values less than 0.05 were considered significant.

## Results

### IL-27 activates STAT1 and STAT3 with resultant translocation to the nucleus in human NSCLC cells

**Figure 1:**
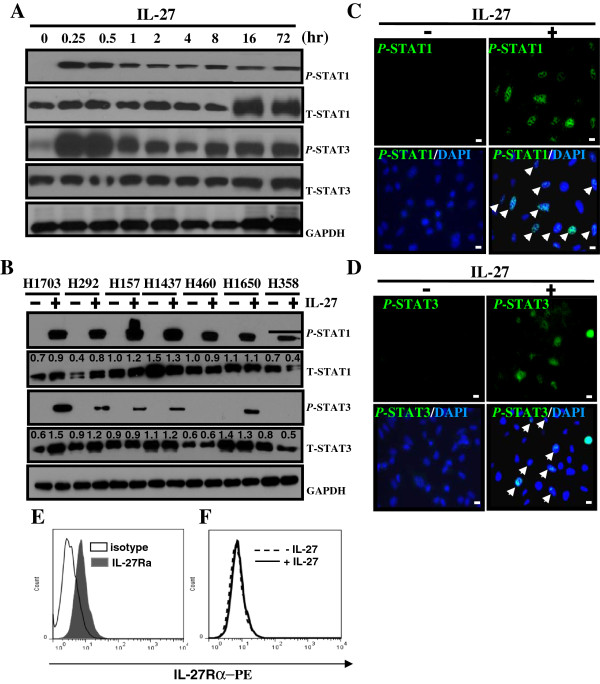
**IL-27-mediated activation of STAT1 and STAT3. (A)** A549 cells were treated with IL-27 (50 ng/mL) for up to 72 hours. The tyrosine phosphorylated, or activated, forms of STAT1 and STAT3 (*P*-STAT1 and *P*-STAT3) as well as the total amounts of the transcriptional factors (T-STAT1 and T-STAT3) were detected by Western blot. **(B)** Seven human NSCLC cell lines (H1703, H292, H157, H1437, H460, H1650, and H358) were cultured with the diluent of IL-27 (0.1% PBS/BSA) or IL-27 (50 ng/mL) for 24 hours and the activated and total amounts of STAT1 and STAT3 proteins were measured by Western blot. The densitometric measurements of total amounts of STAT1 and STAT3 were taken using Image J1.45o. The values above the figures represent relative density of the bands normalized to GAPDH. **(C-D)** A549 cells were treated with IL-27 (50 ng/mL) for 15 minutes, and stained with anti-tyrosine phosphorylated STAT1 **(C)** (green) and STAT3 **(D)** (green) antibodies for immunofluorescence microscopy (50 × magnification). The cells were counterstained with DAPI (blue). The white arrows indicate cells with nuclear activation of STAT1 or STAT3 by IL-27 treatment. Scale bar, 100 μm. **(E)** Expression of IL-27 receptor (TCCR) on cultured A549 cells. **(F)** Expression of IL-27 receptor (TCCR) on A549 cells after treatment with or without IL-27 (50 ng/mL) for 24 hours.

The human lung adenocarcinoma cell line, A549, was treated with IL-27 at time points from 0.25 to 72 hours and analyzed for activated or tyrosine phosphorylated STAT1 (*P*-STAT1) and STAT3 (*P*-STAT3) proteins by Western blot. After addition of IL-27, activation of STAT proteins was observed within 15 minutes with sustained activation for up to 72 hours (Figure 1A). Total STAT1 (T-STAT1) and STAT3 (T-STAT3) levels were not significantly affected by IL-27 exposure.

To validate this concept in other histological subtypes of NSCLC, seven additional human lung cancer cell lines (H1703, H292, H157, H1437, H460, H1650, and H358) were exposed to IL-27 for 24 hours and *P*-STAT1 and *P*-STAT3 protein levels were analyzed by Western blot. Similar to A549 cells, all cell lines, with the exception of H460 and H358, demonstrated activation of both transcriptional factors *P*-STAT1 and *P*-STAT3 following IL-27 stimulation (Figure 1B). Total STAT1 and STAT3 levels were comparable in H157, H1437, H460 and H358 cells. There were increased levels of total STAT1 and STAT3 in H1703 and H292, while decreased in H358 cells, The basis for differential expression of the total STATs in response IL-27 stimulation in lung cancer cells is unclear, but may be related to known underlying mutational heterogeneity of different cancer cell lines [28].

The tyrosine phosphorylated forms of STAT transcriptional factors are known to translocate to the nucleus for regulation of gene transcription [23]. Immunofluorescence microscopy further confirmed STAT1 (Figure 1C) and STAT3 (Figure 1D) protein activation and nuclear translocation in A549 cells. In the absence of IL-27, there were no detectable levels of phosphorylated STAT1 or STAT3 in A549 cells (upper left, Figure 1C and 1D). In contrast, IL-27-treated A549 cells showed phosphorylation of STAT1 and STAT3 following 15 minutes of exposure to IL-27 (upper right, Figure 1C and 1D), with translocation into the nucleus as demonstrated by the overlay of FITC and DAPI staining (bottom right, Figure 1C and 1D).

Next, we tested whether IL-27 treatment affects expression levels of the IL-27 receptor on A549 cells. FACS analysis of A549 cells showed that these cells express substantial amounts of IL-27 receptor (TCCR) on the cell surface (Figure 1E). However, the presence of IL-27 did not affect expression levels of IL-27 receptor on A549 cells at 24 hours (Figure 1F). Evaluation for IL-27 receptor expression at earlier time points (15 minutes, 30 minutes, 1 hour, and 2 hours) was not changed by IL-27 stimulation (data not shown). These results demonstrate that IL-27 activates STAT1 and STAT3 with resultant translocation into the nucleus without altering expression levels of the IL-27 receptor.

### IL-27-mediated STAT activation requires JAK activation

**Figure 2:**
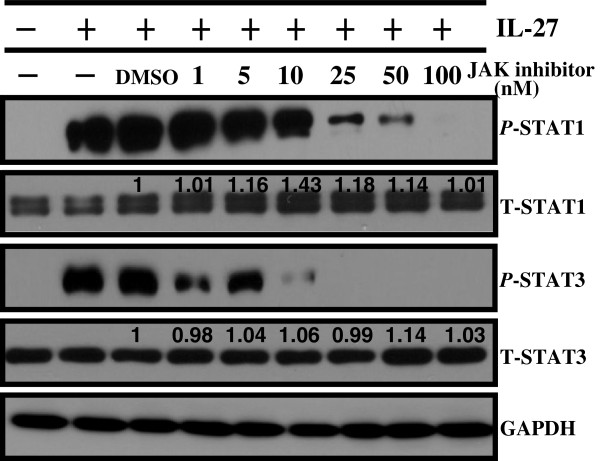
**JAK-dependent activation of STAT1 and STAT3 by IL-27 treatment.** A549 cells were cultured in the presence of JAK inhibitor I (1-100 nM) for 1 hour prior to IL-27 (50 ng/mL) exposure for 24 hours. The activated and total amounts of STAT1 and STAT3 proteins were detected by Western blot. The densitometric measurements of total amounts of STAT1 and STAT3 were taken using Image J1.45o. The values above the figures represent relative density of the bands compared to control DMSO that was set to 1 after normalized to GAPDH.

IL-27 binds a receptor comprised of gp130 and WSX-1, whose intracellular components associate with cytoplasmic protein kinases such as JAKs that mediate cytokine signaling [1]. Upon ligand binding, activated JAKs phosphorylate the receptor and provide docking sites for inactive STAT monomers. The STAT transcriptional factors become phosphorylated by the JAKs, dissociate from the receptor, and dimerize for nuclear translocation [23]. Thus, the importance of JAK signal transduction in the ability of IL-27 to activate the STAT1 and STAT3 pathways in human lung cancer was studied. A549 cells were pre-treated with the vehicle control (DMSO) or a JAK inhibitor for 1 hour followed by exposure to IL-27 and tyrosine phosphorylation of STAT1 and STAT3 proteins was assessed by Western blot. Pre-treatment with the JAK inhibitor resulted in a dose-dependent inhibition of IL-27-mediated STAT1 and STAT3 activation (P-STAT) with a slightly increased expression of the total STAT1 at 5, 10, 25, and 50 nM (Figure 2). In addition, the activation of STAT1 and STAT3 proteins by IL-27 treatment was abolished by pretreatment of cells with the JAK inhibitor, with doses of 100 nM and 25 nM, respectively. IL-27 did not alter the activation of other pathways, including Akt, STAT5, P38, or MAPK/ERK between 15 minutes and 1 hour following treatment of A549 cells (see Additional file 1). These data indicate that JAK activation is required for IL-27-mediated STAT1 and STAT3 activation.

### IL-27 regulates and prevents over-expression of STAT3 through activation of the STAT1 pathway

The specificity of STAT activation is determined by the presence of the docking sites on the receptor, and STAT1 and STAT3 have been shown to be activated in response to gp130 receptor activation by various stimuli [29,30]. STAT1 and STAT3 are known to regulate transcription of target genes playing opposing roles in tumorigenesis [11]. In order to determine if a dominant STAT pathway becomes activated by IL-27, we performed selective inhibition of the STAT1 or STAT3 pathways.

**Figure 3:**
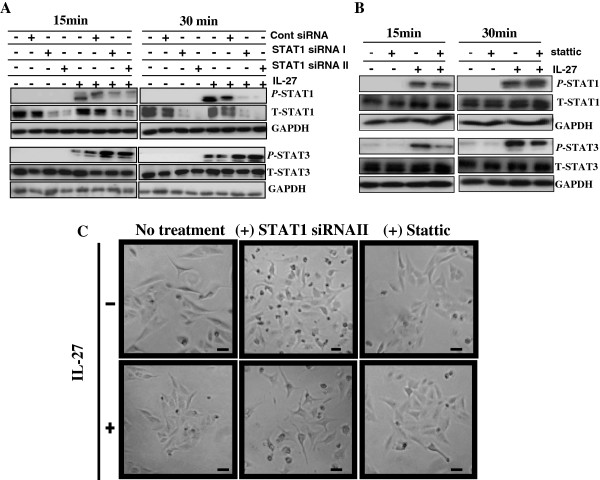
**Acquisition of a more epithelial phenotype by inhibition of STAT1 expression in IL-27 treated cells. (A)** A549 cells were transfected with a non-targeting control or STAT1 siRNAs (40 nM) for 6 hours prior to IL-27 (50 ng/mL) exposure for 15 or 30 minutes. Activated and total amounts of STAT1 and STAT3 proteins were detected by Western blot. GAPDH was used as a loading control. **(B)** Stattic (7.5 nM) or its diluent (DMSO) was added to A549 cells for 1 hour prior to IL-27 (50 ng/mL) exposure for 15 or 30 minutes. Activated and total amounts of STAT1 and STAT3 proteins were detected by Western blot. **(C)** After transfection with STAT1 siRNA (40 nM) for 6 hours or Stattic (7.5 nM) pre-treatment for 1 hour, A549 cells were exposed to IL-27 (50 ng/mL) for 24 hours. Morphologic changes were documented and photographed by phase contrast microscopy (50 × magnification). Scale bar, 100 μm.

A549 cells were transfected with STAT1 siRNAs for 24 hours prior to IL-27 exposure for 15 or 30 minutes, and the activated and total forms of STAT1 and STAT3 were measured by Western blot. The expression of *P*-STAT1 and T-STAT1 proteins was effectively abolished after treatment with STAT1 siRNA I or STAT1 siRNA II while transfection with control siRNA did not significantly affect the level of *P*-STAT1 and T-STAT1 proteins (Figure 3A). It should be noted that lost or reduced p-STAT3 was shown in Figure 3A compared to Figure 1A. This may be due to the procedure of transfection that has been known to induce cellular stress response [31]. Importantly, inhibition of STAT1 resulted in a marked reciprocal increase in *P*-STAT3 compared to control siRNA-transfected cells. It has been previously shown that STAT3 is constitutively activated in A549 cells [32]. Our data suggest that STAT1 protein appears to play an important role in suppressing the overexpression of tyrosine phosphorylated STAT3 in human NSCLC cells.

Given the interdependence of STAT1 and STAT3 activation following IL-27 stimulation, STAT3 inhibition was evaluated by adding Stattic, a nonpeptidic small molecule that inhibits the function of the SH2 domain required for tyrosine phosphorylation, dimerization and subsequent nuclear translocation of STAT3 [33]. The STAT3 inhibitor was added to A549 cells for 1 hour prior to IL-27 exposure for 15 or 30 minutes and the expression of activated and total amounts of STAT1 and STAT3 proteins were analyzed by Western blot. As expected, the expression of *P*-STAT3 was markedly reduced by pretreatment of STAT3 inhibitor at both time points of IL-27 treatment without affecting T-STAT3 (Figure 3B). However, activated or total amount of STAT1 protein was not significantly changed in the pre-treated cells with Stattic when compared with untreated cells, indicating that inhibition of STAT3 alone does not have a considerable impact on STAT1 activation. These results suggest that although IL-27 activates both STAT1 and STAT3, the regulation and prevention of over-expressing phosphorylated STAT3 requires the presence of activated STAT1 in NSCLC cells.

### IL-27 induces an epithelial phenotype in lung cancer cells through STAT1 activation

A fundamental event during EMT is the loss of cell polarity, resulting in transition of polarized epithelial cells into mobile mesenchymal cells [34]. To evaluate the phenotypic changes of NSCLC cells in response to differential STAT1 and STAT3 activation following IL-27 treatment, changes in morphologic features of lung cancer cells were assessed. In comparison to untreated cells (upper left, Figure 3C), IL-27-treated cells exhibited a more epithelial phenotype characterized by a markedly more cohesive and organized appearance of the cells in a cobblestone monolayer formation (lower left, Figure 3C). Suppression of STAT1 expression by siRNA prior to IL-27 treatment resulted in a phenotype characterized by elongated spindle-shaped, fibroblast-like cells that were morphologically similar to untreated cells (lower middle, Figure 3C), while STAT1 siRNA single treatment did not significantly affect the phenotype of untreated cells (upper middle, Figure 3C). The addition of the STAT3 inhibitor (Stattic) did not demonstrate marked morphologic changes in A549 cells when compared to IL-27- treated or -untreated cells (lower right and upper right, Figure 3C). These findings suggest that STAT1 activation is the dominant pathway by which IL-27 mediates polarization of NSCLC cells towards an epithelial phenotype.

### IL-27 promotes expression of epithelial markers through a STAT1 dominant pathway

**Figure 4:**
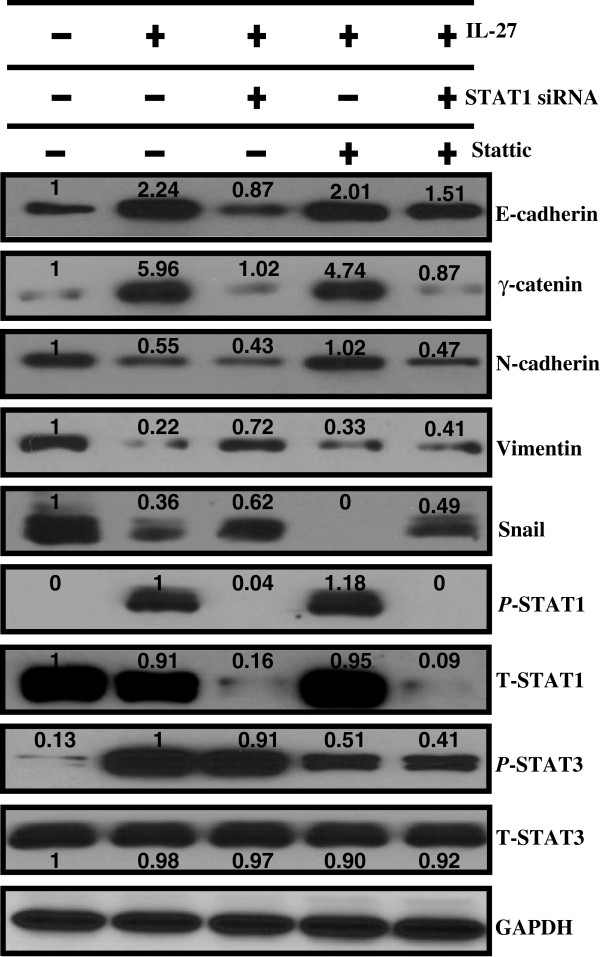
**Increased expression of epithelial and decreased expression of mesenchymal markers by a dominant STAT1 pathway.** After transfection with STAT1 siRNA (40 nM) for 6 hours or Stattic (7.5 nM) pre-treatment for 1 hour, A549 cells were exposed to IL-27 (50 ng/mL) for 24 hours. Proteins responsible for the epithelial phenotype (E-cadherin and γ-catenin) and the mesenchymal phenotype (N-cadherin and vimentin) were detected by Western blot. Changes in Snail levels were also demonstrated by Western blot. Activated and total amounts of STAT1 and STAT3 were also detected, and GAPDH was used as a loading control. Densitometric measurements of the bands were taken using Image J1.45o. The values above the figures represent relative density of the bands normalized to GAPDH.

EMT results in cellular changes associated with alterations in expression of EMT markers [35]. To determine if the STAT1-dependent IL-27 effect on cell morphology correlated with changes in the EMT marker expression, Western blot analysis was performed to examine the expression of E-cadherin and γ-catenin (epithelial phenotype), and N-cadherin and vimentin (mesenchymal phenotype). Snail, a transcriptional repressor of E-cadherin and a key regulator of EMT was also examined [36,37]. Amounts of the activated and total STAT1 and STAT3 proteins were measured along with the EMT markers. IL-27 treated cells showed increased expression of epithelial markers (E-cadherin and γ-catenin) and decreased expression of mesenchymal markers (N-cadherin and vimentin) compared to untreated cells (Figure 4). In addition, the expression of Snail protein was remarkably reduced by IL-27 treatment. These data suggest that IL-27 induces MET.

Next, we examined whether IL-27 induces MET through STAT pathways by blocking STAT1 and STAT3 pathways using STAT1 siRNA or STAT3 inhibitor, Stattic, respectively. As shown in Figure 4, pretreatment with STAT1 siRNA dramatically inhibited expression of T-STAT1, resulting in complete inhibition of STAT1 phosphorylation. Pretreatment with STAT1 siRNA before IL-27 exposure resulted in increased Snail expression, decreased expression of epithelial markers (E-cadherin and γ-catenin), and up regulation of mesenchymal marker (vimentin) compared to treatment with IL-27 alone. STAT1 siRNA mediated down regulation of E-cadherin expression was partially inhibited by the combined treatment with Stattic and STAT1 siRNA given the increased E-cadherin expression when comparing IL-27 + STAT1 siRNA vs. IL-27 + STAT1 siRNA + Stattic groups (Figure 4). These findings suggest that Stattic may directly attenuate the STAT1 siRNA effect on E-cadherin expression. As expected, the total amount of STAT3 protein (T-STAT3) was not changed by Stattic, an inhibitor of STAT3 phosphorylation, but STAT3 phosphorylation was remarkably decreased (Figure 4). When compared to treatment with IL-27 alone, pretreatment with Stattic before IL-27 stimulation did not affect expression of epithelial markers (E-cadherin and γ-catenin) and mesenchymal marker (vimentin), suggesting that STAT1 pathway plays a critical role in the IL-27 mediated regulation of EMT.

Interestingly, there was no significant change in the expression of mesenchymal marker, N-cadherin, by STAT1 inhibition (STAT1 siRNA pretreatment) and the reduced expression of N-cadherin by IL-27 was reversed by STAT3 inhibition (pretreatment with STAT3 inhibitor) (Figure 4), indicating that the decreased expression of N-cadherin by IL-27 may be mediated by STAT3 activation. The decreased expression of Snail by IL-27 was not reversed by inhibition of STAT3 activation.

The mechanism driving the differential effect of IL-27 on the two mesenchymal markers (N-cadherin and Vimentin) is unclear as selective inhibition of STAT1 or STAT3 did not elucidate a clear mechanism (Figure 4). Instead, there was suggestion that STAT3 may be involved in N-cadherin expression (Figure 4). Although N-cadherin is considered a mesenchymal marker, its function may be more complex as other studies have shown that repression of N-cadherin is required for epithelial to mesenchymal transition in some instances such as neural crest migration [34,38]. However, the overall effect with IL-27 stimulation in our study was promotion of mesenchymal to epithelial transition. The impact of N-cadherin and STAT3 in this process is unclear.

Overall, these results suggest that the STAT3 pathway is not critically involved in the IL-27 mediated promotion of epithelial marker expression. In summary, STAT1 appears to be the dominant pathway by which IL-27 promotes the expression of epithelial markers.

Of note, the reciprocal increase in P-STAT3 compared to control with inhibition of STAT1 by siRNA seen in Figure 3A is not demonstrated in Figure 4. These are two different experiments where the duration of IL-27 stimulation and time point for measurement of P-STAT3 expression are entirely different for the two figures.

### IL-27 inhibition of in vitro cell migration is mediated by a STAT3-independent and STAT1-dependent pathway

**Figure 5:**
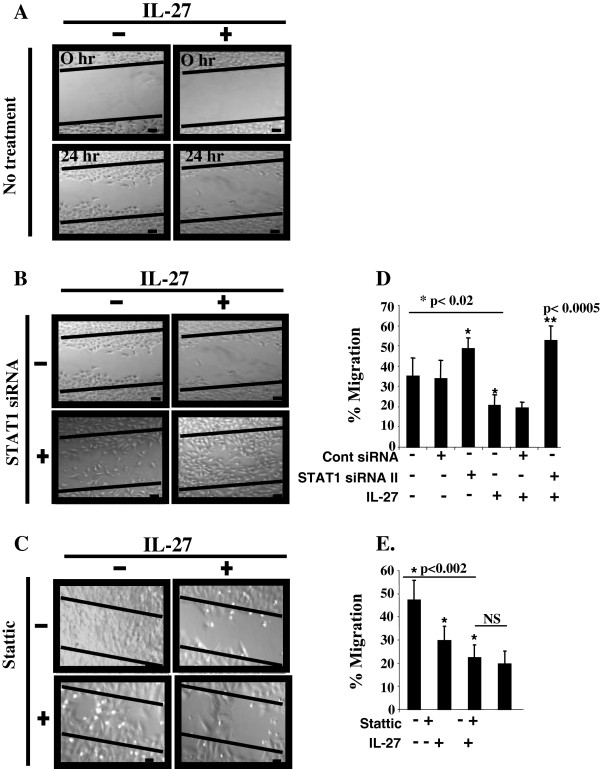
**Inhibition of *in vitro *cell migration dependent upon STAT1 activation. (A)** A549 cells were treated with IL-27 (50 ng/mL) at 60 ~ 70% confluency for 24 hours and a scratch was created in the cell monolayer. The same fields were observed for cell migration using phase contrast microscopy after 24 hours of IL-27 treatment. **(B)** The scratch technique was utilized to measure cell migration for A549 cells that were transfected with STAT1 siRNA (40 nM) for 24 hours prior to with or without IL-27 (50 ng/mL) exposure. **(C)** The motility assay was employed to measure cell migration after Stattic (7.5 nM) pre-treatment for 1 hour prior to IL-27 exposure (50 ng/mL), and changes in cell migration were observed for 60 hours. Scale bar, 200 μm. **(D and E)** Cell migration evaluated using transwell chambers. A549 sells transfected with STAT siRNAs for 24 hrs, control siRNA-transfected or untreated cells **(D)** followed by 1 hour of Stattic treatment **(E)** were plated 24 h after treatment with IL-27 on 96-well transwell plates. After 48 hours, cells that migrated through the pores to the under surface of the membrane and bottom wells were labeled with Calcein-AM. Migration rate was calculated using fluorescence as described in Materials and Methods.

To further evaluate phenotypic changes associated with IL-27- epithelial marker expression beyond morphologic appearance, we examined *in vitro* cell migration, a defining feature of the mesenchymal phenotype, by creating a scratch or wound in a confluent monolayer of NSCLC cells and evaluating wound closure as a result of cell migration. Borders of the wound were marked by solid black lines. We expected IL-27 to inhibit cell migration through STAT1 pathway. Indeed, A549 cells treated with IL-27 showed only poor migration into the border line (lower right, Figure 5A) whereas untreated cells displayed rapid migration after 24 hours of IL-27 treatment (lower left, Figure 5A). Next, we examined whether the inhibitory effect of IL-27 on migration is related to STAT pathways using STAT1 siRNA and STAT3 inhibitor, Stattic. Again, whereas untreated cells demonstrated rapid cell migration toward each other with partial closing of the gap between the solid black lines (upper left, Figure 5B), IL-27 treated cells showed remarkably decreased cell migration (upper right, Figure 5B). Pretreated cells with STAT1 siRNA showed no significant difference in cell migration as compared to untreated cells (lower left, Figure 5B). However, pretreatment with STAT1 siRNA prior to IL-27 exposure caused a marked increase in cell migration compared to untreated cells, and reversed the inhibitory effect of IL-27 on cell migration as demonstrated by the near complete wound closure between the black lines (lower right, Figure 5B), suggesting that STAT1 is required for the inhibitory effect of IL-27 on cell migration. We also evaluated the inhibition of the STAT3 pathway before IL-27 exposure using a STAT3 inhibitor, Stattic. IL-27-treated cells still maintained a large gap between the solid black lines (upper right, Figure 5C) when compared to untreated cells that closed the gap created by the scratch after 60 hours of IL-27 treatment (upper left, Figure 5C). The addition of the STAT3 inhibitor did not significantly affect the inhibitory effect of IL-27 on migration (lower right, Figure 5C), suggesting that IL-27 mediated inhibition of cell migration may not be dependent on STAT3 activation.

Cell migration was further studied using the transwell chamber migration assay in which the results were consistent with scratch/wound assay findings. The addition of IL-27 inhibited transwell cell migration (Figure 5D). Treatment with STAT1 siRNA with or without IL-27 significantly increased transwell cell migration compared to control siRNA group (Figure 5D). As such, STAT1 siRNA prevented IL-27 mediated inhibition of cell migration. In contrast, the addition of Stattic showed a significant inhibition of cell migration (Figure 5E). Taken together, our results demonstrate that IL-27 inhibits *in vitro* cell migration via a STAT1 dependent mechanism and that STAT3 does not appear to be essential in the inhibitory effect. 

### IL-27-mediated inhibition of angiogenic factors is STAT1-dependent

Tumor growth and metastasis are integrally dependent on production of angiogenic factors and angiogenesis [39-41]. Vascular endothelial growth factor (VEGF) is well known potent angiogenic factor [42]. In addition to VEGF, IL-8/CXCL8 and CXCL5 have been identified as important pro-angiogenic proteins in human NSCLC [43,44]. It has previously been shown that IL-27 has anti-angiogenic activity by down regulating the expression of VEGF, IL-8/CXCL8 and CXCL5 in human multiple myeloma cells [3]. In this study, we examined the production of pro-angiogenic factors, VEGF, IL-8/CXCL8, and CXCL5, to determine the effects of IL-27 on angiogenesis in human lung cancer.

STAT1 and STAT3 are known to have opposing roles in VEGF regulation. For example, STAT1 has been shown to be a negative regulator of VEGF and angiogenesis [16,45,46]. In contrast, STAT3 transactivation with other factors is required for full induction of the VEGF promoter in cancer cells [47]. Similarly, STAT1 is required for inhibition of IL-8 expression mediated by other cytokines [48]. Constitutive activation or knockdown of STAT3 has been shown to up regulate or suppress IL-8 production in human melanoma cells, respectively [49]. The role of STAT1 and STAT3 pathways in the production of CXCL5 in cancer has not been well studied.

**Figure 6:**
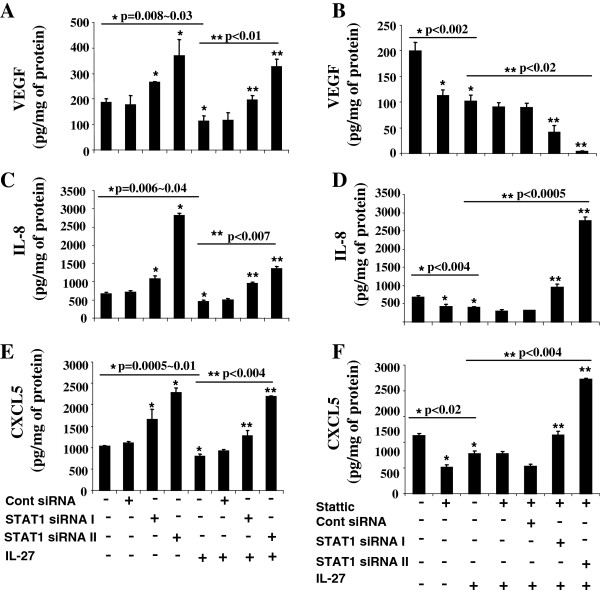
**Down-regulation of angiogenic factors and up-regulation of angiostatic factors by STAT1-dependent pathway. (A-F)** Protein concentrations of VEGF **(A, B)**, IL-8/CXCL8 **(C, D)**, CXCL5 **(E, F)** secreted by A549 cells were measured by ELISA. A549 cells were either transfected with STAT1 siRNAs (40 nM) or control siRNA for 24 hours and further treated with or without Stattic (7.5 nM) for 1 hour followed by IL-27 (50 ng/mL) treatment for 24 hours. The cell culture supernatants were used for ELISA. * p vs. no treatment, ** p vs. IL-27 by student *t-* test.

On this basis, the expression of angiogenic factors were measured in A549 cells by ELISA after being exposed for 24 hours to IL-27 alone or after being pre-treated with STAT1 siRNA or STAT3 inhibitor, Stattic. Our results demonstrate that the inhibition of STAT1 by siRNA in A549 cells led to increased production of VEGF, IL-8 and CXCL5 (Figure 6A, 6C, and 6E) while the suppression of STAT3 activation caused reduced secretion of the pro-angiogenic factors (Figure 6B, 6D, and 6F). IL-27 treated cells showed statistically significant decrease in expression of VEGF, IL-8/CXCL8, and CXCL5 compared to untreated cells (Figure 6A, 6C, and 6E, respectively). Inhibition of the STAT1 pathway by pretreatment with STAT1 siRNA, but not control siRNA, reversed the IL-27 mediated decreased expression of VEGF, IL-8/CXCL8, and CXCL5, resulting in increased levels of these pro-angiogenic factors to levels significantly higher than untreated controls.

The impact of the STAT3 pathway was also studied by the addition of Stattic to the IL-27-treated cells. Pretreatment with the inhibitor of STAT3 activation did not reverse the inhibitory effect of IL-27 on the production of VEGF, IL-8/CXCL8, and CXCL5, but rather led to further decrease in the production of IL-8/CXCL8 when compared to IL-27 alone (Figure 6B, 6D, and 6F), suggesting that IL-27 mediated inhibitory effect on the production of pro-angiogenic factors associated with angiogenesis is independent of STAT3.

Inhibition of both STAT1 and STAT3 activation also reversed the reduction of IL-8 and CXCL5 by IL-27 treatment as demonstrated by the significantly increased expression compared to IL-27 alone (Figure 6D and 6F). The combined STAT1 and STAT3 inhibition effect of reciprocal increased IL-8 and CXCL5 levels did not impact VEGF (Figure 6B). These results suggest that STAT1-dependent inhibitory effect of IL-27 on the production of VEGF may also require STAT3 activation.

Overall, our findings support that STAT1, but not STAT3, plays a primary role in inhibition of pro-angiogenic factor production in human lung cancer by IL-27 treatment. Furthermore, inhibition of STAT1 results in augmentation of pro-angiogenic factors beyond the basal level possibly due to increased STAT3 activation in addition to STAT1 inhibition as shown in Figure 3A. Our data suggests that the impact of basal STAT1 expression may regulate STAT3 activation to control angiogenesis.

## Discussion

Epithelial to mesenchymal transition and angiogenesis have emerged as integral processes in promoting carcinogenesis [50]. The change from epithelial to mesenchymal phenotype has been associated with tumor invasion, metastasis, and unfavorable prognosis [51]. The role of STAT pathways in regulating EMT during carcinogenesis and embryogenesis has been described in a limited number of studies. For example, STAT1 and STAT5 have been shown to be involved in regulating EMT during renal tubule formation and in mammary gland growth and epithelial differentiation, respectively [52,53]. In cancer, STAT3 has been implicated in EGF-mediated EMT in ovarian cancer cell lines and STAT1 has been reported to inhibit angiogenesis in murine fibrosarcoma tumor cells [16].

Epithelial and mesenchymal marker expression is known to be important in EMT. Factors such as E-cadherin, catenins, vimentin, and snail have all been correlated with clinical and pathological features in non-small-cell lung cancer [54-56]. Transcriptional repression of E-cadherin by Snail is closely correlated with EMT and the loss of E-cadherin expression is a hallmark of EMT [57]. The expression of E-cadherin and catenins is reduced in NSCLC [55,56]. In addition, vimentin is over-expressed in many epithelial cancers, including lung cancer, and its over expression in cancer correlates with tumor growth, invasion, and poor prognosis [58].

IL-27 has been shown to have non-immune antitumor effects in lung cancer that include suppression of COX-2 and PGE2, reduction of vimentin levels, and inhibition of cell migration and invasion [27]. This study showed that IL-27 treatment in lung cancer cells led to increased E-cadherin expression and decreased expression of vimentin and Snail with inhibition of cell migration by suppression of cyclooxygenase-2-mediated activities [27].

The importance of IL-27 in modulating EMT through the STAT pathways is poorly understood in carcinogenesis. To our knowledge, there have been no studies that have described MET as an anti-tumor mechanism of IL-27. In our study, we hypothesized that IL-27 inhibits EMT and angiogenesis through STAT dependent pathways.

Our results revealed that IL-27-treated lung cancer cells show increased epithelial marker (E-cadherin and γ-catenin), decreased Snail (transcriptional repressor of E-cadherin), and decreased mesenchymal marker (N-cadherin and vimentin) expression. In addition, IL-27 treatment suppressed *in vitro* cell migration. The ability of IL-27 to promote MET and inhibit cell migration was abolished by inhibition of the STAT1 pathway, but not the STAT3 pathway, with the exception of N-cadherin expression. The impact of N-cadherin and STAT3 in this process is unclear.

Overall, our findings suggest that IL-27 promotes MET and the increased expression of epithelial marker proteins is STAT1-dependent. The inhibition of EMT through STAT1 dependence is a novel anti-tumor mechanism of IL-27, which has not been previously described.

Our results support the body of evidence that STAT1 is associated with tumor suppressive properties, such as inhibition of angiogenesis, tumor growth and metastasis as well as promotion of apoptosis [12,16]. The role of STAT3 in IL-27 regulation of EMT is not well understood. In present study, the inhibition of STAT3 activation did not reverse the increased expression of epithelial markers (E-cadherin and γ-catenin) and the reduced expression of mesenchymal marker (vimentin) and Snail by IL-27, and STAT3 activation was not required for the inhibition of cell migration by IL-27. Interestingly, the inhibition of STAT1 activation led to increased STAT3 activation in IL-27 treated lung cancer cells whereas inhibition of STAT3 activation alone did not significantly impact STAT1 expression. The current study does not provide a mechanism by which inhibition of STAT1 led to increased STAT3 activation. However, similar to our results, previous studies have demonstrated that STAT1- deficient cells showed increased STAT3 activation [59-61]. Potential mechanisms by which STAT1 may directly inhibit STAT3 include competition for receptor docking sites, promoters of target DNA sequences, and/or binding cofactors. The receptor docking site is a prerequisite for activation by tyrosine phosphorylation and STAT3 can be phosphorylated by receptor bound tyrosine kinases [62,63]. In fact, it has been shown that STAT1 suppresses STAT3 tyrosine phosphorylation that mediates downstream signaling of other cytokine receptors [60]. Thus it appears likely that STAT1 suppresses IL27-mediated STAT3 activation at least in part by competing for the STAT docking site within the IL-27 receptor cytoplasmic domain.

Our results also demonstrated that the inhibition of STAT1 pathway in IL-27 treated cells resulted in augmented cell migration and increased production of pro-angiogenic factors (VEGF, IL-8, and CXCL-5) compared to untreated cells. These findings may be due to the enhanced STAT3 activation in the setting of inhibition of STAT1 activation. Activated STAT3 has been shown to play an important role in oncogenic transformation and progression in many human cancers [13-15,17-20]. STAT3 has been shown to regulate cell migration, motility and invasion [64-66] and induce VEGF expression [18]. The anti-angiogenesis properties of IL-27 in tumor models have been described previously. It has been shown that anti-tumor and anti-angiogenic activities of IL-27 in murine melanoma tumors [5]. Cocco *et al.* described anti-angiogenic properties of IL-27 in a multiple myeloma tumor model [3]. However, these studies did not define the mechanism of IL-27 mediated inhibition of angiogenesis. The augmented cell migration and promotion of angiogenesis factors may be due to the reciprocal increase of STAT3 activation in the setting of STAT1 inhibition. This hypothesis of STAT1 and STAT3 interdependence is further supported by other reports using a genomic technique to map transcriptional factor binding sites and identified STAT3 as a direct transcriptional target of STAT1 [67]. It has also been shown that STAT3 was activated in a sustained strong manner in STAT1 knock-out murine fibroblasts [60,68]. On this basis, basal STAT1 activation may be required in repressing STAT3 activation.

Cytokines, such as IL-27, that possess divergent functions may play a pivotal role in influencing immune regulation and carcinogenesis through differential STAT1 and STAT3 activation and cross-regulation. There have been limited reports understanding the regulation of EMT in carcinogenesis through STAT pathways. Although the anti-tumor properties of IL-27 have been described previously, our study describes a new mechanism by which IL-27 inhibits EMT and angiogenesis through a STAT1 dominant pathway.

## Conclusions

We report that IL-27-mediated induction of MET and inhibition of angiogenic factors is STAT1-dependent, and inhibition of STAT1 activity results in induction of a mesenchymal phenotype and angiogenic factors above basal levels implicating an overwhelming STAT3 effect. These findings suggest that STAT1 activation may play an important role in repressing STAT3 in lung carcinogenesis, and suggest that better understanding of STAT signaling by cytokines such as IL-27 may shed light to potential new targets in cancer prevention and therapy.

## Competing interests

The authors declare that they have no competing interests.

## Authors’ contributions

PK, LZ, MHL, SMD, and JML are responsible for the study design. PK, LZ and MHL, performed the experiments and collected the data. PK, LZ, MHL, FB, GL, MS, GW, SS, SMD, and JML participated in the data analysis and interpretation. PK, MHL, TCW, KK, SMD, and JML drafted the manuscript. All authors read and approved the final manuscript.

## Supplementary Material

Additional file 1 **IL-27 did not alter the activation of other signaling pathways.** A549 cells were treated with IL-27 (50 ng/mL) for 15 minutes to 1 hour. The phosphorylated forms of Akt, STAT5, p38 and MAPK/ERK1/2 were detected by Western blot. Click here for file
