# Supplementary figures and images for: IL-27 inhibits epithelial-mesenchymal transition and angiogenic factor production in a STAT1-dominant pathway in human non-small cell lung cancer
Source: J Exp Clin Cancer Res. 2013 Nov 25;32(1):97. doi: 10.1186/1756-9966-32-97 (PMC3906956; doi:10.1186/1756-9966-32-97)

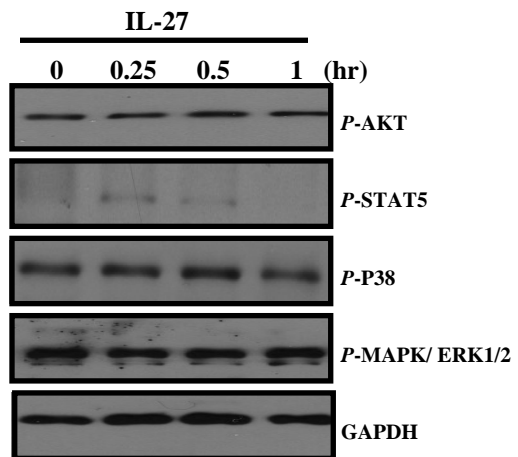

**Additional file 1. Kachroo, P. et al.**

Supplement: Additional file 1 — IL-27 did not alter the activation of other signaling pathways. A549 cells were treated with IL-27 (50 ng/mL) for 15 minutes to 1 hour. The phosphorylated forms of Akt, STAT5, p38 and MAPK/ERK1/2 were detected by Western blot. [file 1756-9966-32-97-S1.pdf]
